# Halitose: approches diagnostiques et thérapeutiques pluridisciplinaires

**DOI:** 10.11604/pamj.2018.30.201.10951

**Published:** 2018-07-10

**Authors:** Roger Sombié, Arnaud Jean Florent Tiendrébéogo, Wendpouiré Patrice Laurent Guiguimdé, Alice Guingané, Souhouto Tiendrébéogo, Kampadilemba Ouoba, Alain Bougouma

**Affiliations:** 1 Service d’Hépato-gastroentérologie, Centre Hospitalo-Universitaire Yalgado Ouédraogo, Burkina Faso; 2 Service de Pneumologie, Centre Hospitalo-Universitaire Yalgado Ouédraogo, Burkina Faso; 3 Service de Chirurgie Dentaire, Centre Hospitalo-Universitaire Yalgado Ouédraogo, Burkina Faso; 4 Service d’Oto-rhino-laryngologie et de Chirurgie Cervico-faciale, Centre Hospitalo-Universitaire Yalgado Ouédraogo, Burkina Faso

**Keywords:** Halitose, diagnostic, traitement, H.pylori, Halitosis, diagnosis, treatmenT, H.pylori

## Abstract

**Introduction:**

**L**’halitose, état morbide caractérisé par une mauvaise haleine, présente à la fois un aspect pathologique et social. Dans notre contexte, l’halitose pose de nombreux problèmes de prise en charge aux plans diagnostique et thérapeutique en pratique clinique. Le but de notre travail était d’étudier les aspects diagnostiques et thérapeutiques de l’halitose.

**Méthodes:**

Il s’agit d’une étude transversale sur une année. Ont été inclus, les patients âgés de plus de 15 ans qui ont consulté pour halitose au centre hospitalier universitaire Yalgado Ouédraogo. Ont été exclus les patients avec mauvaise haleine mais consultant pour un autre motif. L’haleine a été évaluée par un praticien selon le test organoleptique de Rosenberg.

**Résultats:**

Au total 35 patients ont été inclus pour un sex-ratio de 1,2. L’âge moyen était de 31,9 ans. Dans 57,1% des cas, la plainte venait du patient lui-même. La durée moyenne de l’halitose était de 4,3 ans. Dix-neuf patients avaient un score de Mel Rosenberg ≥ 2. La carie dentaire (07 cas), la sinusite (07 cas), l’infection à *Helicobacter pylori* (09 cas) et l’ulcère gastro-intestinal (10 cas) étaient associés à l’halitose. Le traitement a été étiologique dans 82.9% des cas avec une amélioration satisfaisante à deux semaines de l’ordre de 71,8%.

**Conclusion:**

L’halitose reste une pathologie peu étudiée et pose un problème de diagnostic positif, mais aussi étiologique dans notre contexte. Le rôle de l’odontologiste est crucial dans la recherche de la cause de l’halitose. Cependant, une prise en soins pluridisciplinaire de l’halitose permettra d’y apporter une réponse plus efficace.

## Abstract (en)

**Introduction:**

Halitosis is a morbid condition characterized by bad breath. Sometimes it has pathological and social implications. In the context of daily clinical practice, halitosis poses many diagnostic and therapeutic problems. This study aims to investigate the clinical and therapeutic features of this disorder.

**Methods:**

We conducted a cross-sectional study over a one year period. Patients over the age of 15 years presenting with halitosis at the University Hospital Yalgado Ouedraogo were enrolled in the study. Patients with bad breath but presenting with some other problema were excluded. Patient’s breath was assessed by a practitioner on the basis of Rosenberg organoleptic test.

**Results:**

A total of 35 patients were included, with a sex ratio of 1.2. The average age of patients was 31.9 years. In 57.1% of cases, complaints were made by patients themselves. The mean duration of halitosis was 4.3 years. Nineteen patients had Mel Rosenberg score ≥ 2. Dental caries (07 cases), sinusitis (07 cases), Helicobacter pylori infection (09 cases) and gastrointestinal ulcer (10 cases) were associated with halitosis. Treatment was based on etiology in 82.9% of cases with satisfactory improvement after two weeks in 71.8% of cases.

**Conclusion:**

Halitosis is a little studied disorder which poses problem in positive diagnosis as well as etiologic problems in our context. The dentist plays a crucial role in identifying the possible cause of halitosis. However, multidisciplinary approach would enable a more effective response.

## Introduction

L’halitose est un état morbide caractérisé par une mauvaise odeur émanant de la cavité buccale, indépendamment de l’origine intra ou extra orale [1,2]. Elle présente à la fois un aspect pathologique et social car peut constituer un sérieux handicap social avec des conséquences psychologiques non négligeables [3-5]. Malgré la difficulté diagnostique probablement liée à l’utilisation de diverses méthodes de mesure de l’halitose, la prévalence de l’halitose est estimée entre 2% et 49% selon les études [6]. L’halitose, aux diverses étiologies (orales et/ou non-orales) pourrait être le reflet d’une pathologie locale ou générale sous-jacente [7]. Les pathologies non-buccales identifiées comme des facteurs induisant une halitose comprennent notamment des pathologies oto-rhino-laryngologiques (ORL), des infections du tractus gastro-intestinal et des voies respiratoires supérieures et inférieures, et certaines maladies métaboliques, psychologiques ou iatrogènes [8,9]. Les pathologies gastro-intestinales incriminées sont essentiellement le reflux gastro-œsophagien, le diverticule de Zenker, l’achalasie, le cancer de l’œsophage, l’ulcère gastroduodénal, la sténose du pylore et l’infection gastrique à *Helicobacter pylori (H. pylori)* [8,10,11]. Au Burkina Faso, le diagnostic et la prise en charge de l’halitose ne font pas encore partie du curriculum ni dentaire ni médical justifiant d’une part les difficultés de diagnostic et de prise en charge thérapeutique de cette maladie et d’autre part l’absence de publication scientifique à notre connaissance. Le but de ce travail était d’étudier les caractéristiques et les aspects diagnostiques et thérapeutiques de l’halitose au Centre Hospitalier Universitaire - Yalgado Ouédraogo (CHU-YO) de Ouagadougou (Burkina Faso).

## Méthodes

Il s’est agi d’une étude transversale qui s’est déroulée du 1^er^ Aout 2013 au 31 Juillet 2014. Ont été inclus dans l’étude selon un échantillonnage de convenance, les patients d’âge supérieur à 15 ans, et consultant d’eux-mêmes pour halitose, dans les services d’hépato-gastro-entérologie, de chirurgie dentaire, de chirurgie maxillo-faciale et d’Oto-Rhino-Laryngologie (ORL) du Centre Hospitalier Universitaire Yalgado Ouédraogo. Les patients présentant une halitose, mais consultant pour un autre motif, n’ont pas été retenus. Dans chaque service hospitalier, un médecin référent réalisait l’examen clinique des patients; après obtention des consentements oraux éclairés des patients ou des parents pour les patients mineurs pour participer à l’étude, il orientait le patient vers le médecin enquêteur. Ce dernier était chargé de réaliser les entretiens directs avec les patients et de collecter les données sur une fiche individuelle à partir des dossiers de consultation, des fiches d’entretien et de suivi.

L’haleine a été évaluée par le praticien consultant selon le test organoleptique de Rosenberg [8]. Pour chaque étape, un score est accordé pour l’air émanant de la bouche ou du nez selon la classification de Rosenberg [12,13]. Une endoscopie digestive haute (proposée à tous), n’était réalisée qu’après le consentement du patient. Les biopsies gastriques à la recherche de *H. pylori*, n’étaient pas systématiques. L’évolution sous traitement a été évaluée sur l’avis du patient, à l’aide d’une échelle verbale simple (pas satisfaisant, modérément satisfaisant, bien satisfaisant) en consultation ou par contact téléphonique une et deux semaines après. Ainsi, pour chaque patient nous avons recueilli des données épidémiologiques, cliniques (antécédents, facteurs de risque et caractéristiques de l’halitose, données de l´examen clinique et de l’évolution clinique), para cliniques et thérapeutiques. Les données ont été ensuite saisies à l´aide du logiciel de traitement Excel 2007; et analysées avec le logiciel SPSS 16.0. Après une analyse descriptive des différentes variables d’intérêts décrites ci-dessus, nous avons utilisé les tests de χ^2^ (Chi-2) pour l’analyse des variables quantitatives puis procédé à une analyse multivariée pour identifier les facteurs prédictifs de gravité de l’halitose (intensité de l’haleine, score de Rosenberg,…). Les résultats ont été considérés comme significatifs pour une probabilité p < 0,05.

## Résultats

Au total 35 patients ont été inclus dont 19 hommes (54,3%), pour un sex-ratio de 1,2. L’âge moyen était de 31,9±1,7 ans (extrêmes à 18 et 59 ans). L’origine de la plainte venait du patient lui-même dans 57,1% des cas (20 patients). L’halitose se manifestait la bouche ouverte (97,1% des cas soit 34 patients) et de façon permanente (60% des cas soit 21 patients) (Tableau 1). Le parfum de la mauvaise haleine, déclaré dans 31% des cas (11 patients), avait une odeur d’aliments pourris (chou, œuf, poisson) dans 20% des cas (7 patients); l’intensité de l’halitose était moyenne dans la 40% des cas soit 14 patients (Tableau 1). La durée moyenne de l’halitose était de 4,3±0,7 ans (extrêmes 1-25 ans). L’halitose générait un stress chez tous les patients et était responsable d’un handicap socioprofessionnel chez 65,7% des cas (23 patients) (Tableau 2). Le chewing-gum était le moyen le plus utilisé pour masquer l’halitose (19 patients soit 54,3%); étaient également utilisés le bonbon (28,6%), les pastilles (11,4%), la pâtisserie (2,9%) et le brossage des dents (2,9%).

**Tableau 1:** Caractéristiques de l’halitose (n=35)

*Caractéristiques de l’halitose*		*Nombre (%)*
*Moment*	Matin	3 (8,6)
	Soir	1 (2,9)
	Permanent	21 (60)
	Jeûne	4 (11,4)
	Non précisé	6 (17,1)
*Parfum*	Aliments pourris	07 (20)
	Selles	04 (11,4)
	Non précisé	23 (65,7)
*Intensité*	Très forte	8 (22,9)
	Moyenne	14 (40)
	Faible	6 (17,1)
	Non précisée	7 (20)

**Tableau 2:** Retentissements socioprofessionnels majeurs de l’halitose (n=25)

*Impact*	*Effectif*	*Attitude de l’entourage*	*Conséquences*
Professionnel	03	Évitement, fuite du regard, mains sur le nez, moqueries.	Refus ou peur de parler, honte, gêne.
Familial ou amical	19		
Conjugal	03	Refus ou inconfort	Divorce, plaintes.

NB: *aucun retentissement socioprofessionnel chez 7 patients et non précisé dans 3 cas*.

**Figure 1:**
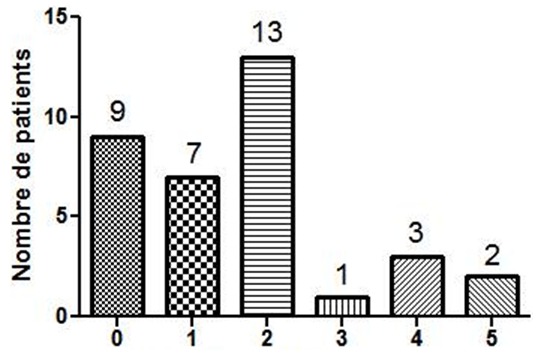
Échelle de Mel Rosenberg

**Figure 2:**
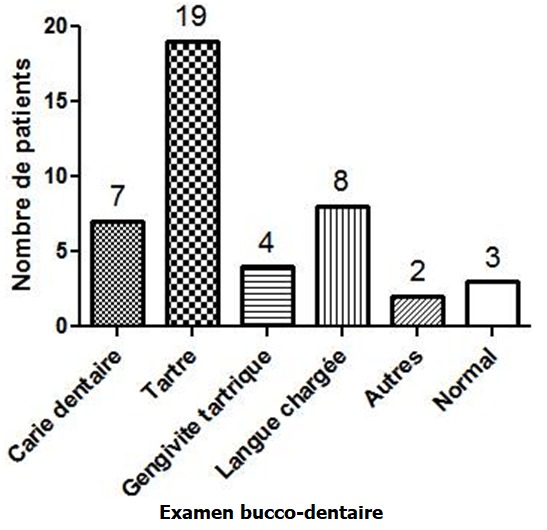
Examen bucco-dentaire des patients atteints d’halitose

**Figure 3:**
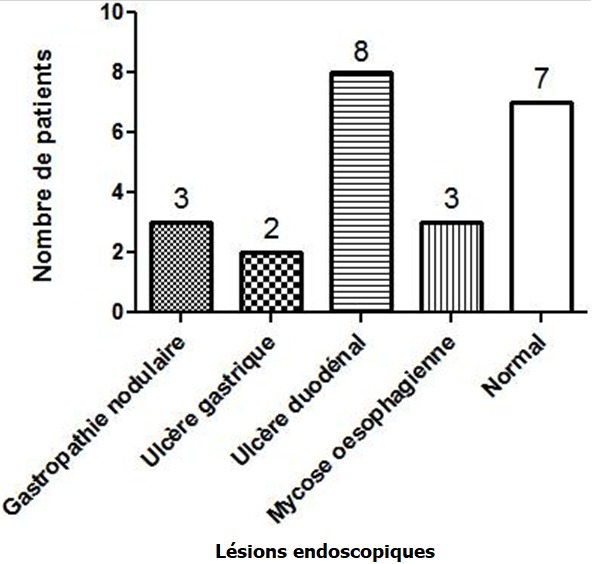
Lésions endoscopiques chez des patients atteints d’halitose

**Figure 4:**
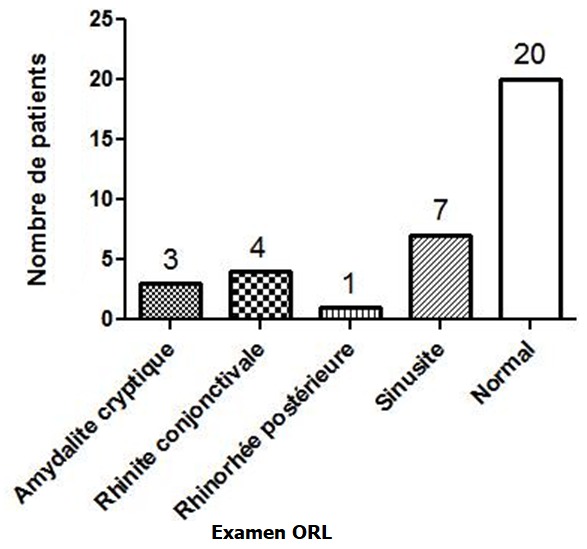
Examen ORL des patients atteints d’halitose

La consultation en chirurgie dentaire et en hépato-gastroentérologie était respectivement de 57,1% et 22,9%. Dix-neuf patients sur 35 (54,3%) avaient un score de Mel Rosenberg ≥ 2 (Figure 1). L’examen bucco-dentaire a trouvé une carie dentaire dans 7 cas (20%), une langue chargée dans 8 cas (22,8%) (Figure 2). Sur le plan digestif, 7 patients (20%) avaient un trouble fonctionnel intestinal (douleurs abdominales, ballonnements, constipation et/ou diarrhée). L’endoscopie digestive haute réalisée chez 21 patients (Figure 3), a trouvé 3 cas de mycose œsophagienne, 8 cas d’ulcère duodénal, 2 cas d’ulcère gastrique et 3 cas de gastropathie nodulaire. *H. pylori* était positif chez 9 patients sur 10 dépistés, chez lesquels une biopsie gastrique a été effectuée. A l’histologie, une gastrite chronique était présente chez tous les patients infectés. L’examen ORL était normal chez 20 patients et notait une sinusite, une amygdalite cryptique dans respectivement 7 cas (20%) et 3 cas (8,6%) (Figure 4). En analyse univariée la carie dentaire était statistiquement associée à l’intensité de l’halitose (p = 0,011) et la sinusite à la durée des symptômes de l’halitose (p = 0,008). En analyse multivariée prenant en compte la carie dentaire, la sinusite, l’ulcère gastroduodénal et les troubles fonctionnels intestinaux, l’association entre la carie dentaire et l’intensité de l’halitose persistait (p = 0,036; IC 95% [0,046-1.242]) et l’ulcère gastroduodénal était négativement associé au score de Rosenberg (p = 0,018; IC 95% [-3.987; -0,399]).

La prise en charge (PEC) des patients était pluridisciplinaire dans 37,1% des cas. Le traitement a été étiologique dans 82,9%. Le traitement symptomatique consistait en des conseils d’hygiène bucco-dentaire ou l’utilisation de cosmétiques dans respectivement 60,9% et 42,9%. Le taux de suivi des patients à une et deux semaines était respectivement de 80% et 91,4%. L’appréciation des patients sur l’amélioration de l’halitose était satisfaisante à deux semaines dans 71,8% des cas (Tableau 3).

**Tableau 3:** Evolution de l’halitose sous traitement

Evolution	Une semaine, n (%)	Deux semaines, n (%)
Pas satisfaisant	08 (28,6)	09 (28,1)
Modérément satisfaisant	17 (60,7)	18 (56,2)
Bien satisfaisant	03 (10,7)	05 (15,6)
Total	28 (100)	32 (100)

## Discussion

L’originalité de ce travail est qu’à notre connaissance, c’est la première étude clinique pluridisciplinaire à porter sur l’halitose dans un pays africain, où l’halitose est une maladie méconnue, honteuse et tabou. Peu de patients consultent en effet pour une halitose à cause de son caractère tabou dans notre contexte, rendant difficile l’évaluation de sa prévalence. Nonobstant ces difficultés, nous avons étudié la fréquence ainsi que les caractéristiques cliniques de l’halitose tout en évaluant le parcours de soin des patients atteints de cette maladie au sein du Centre Hospitalier Universitaire Yalgado Ouédraogo. Notre petit effectif, 35 cas d’halitose sur une année d’activité hospitalière, sous-estime très probablement la fréquence ; et pourrait se justifier d’une part par le fait que l’halitose reste toujours un sujet tabou dans notre pays si bien que les patients ont honte d’en parler et n’ont pas le courage de consulter, d’autre part par la non prise en compte des patients présentant une halitose mais consultant pour un autre motif. Nos résultats sont en accord avec les données de la littérature car les informations concernant la fréquence de l’halitose sont peu abondantes avec 25 à 40% de la population mondiale qui en est atteinte [8]. Elle est estimée à 27,5% en Chine, 24% aux USA et 6 à 23% au Japon [8,14,15]. En Afrique, les données sur l’halitose sont rares; Aka en Côte d’ivoire rapportait 11 patients consultant pour halitose (sur un total d’environ 2000 consultants) durant l’année 1997 [3].

L’halitose, de son diagnostic à son traitement, est en effet un aspect peu développé dans l’exercice médical quotidien au Burkina Faso. Le diagnostic de l’halitose, comme pour toute maladie, est le résultat de l’interrogatoire, des examens clinique et para clinique [5]. L’évaluation de la présence d’une mauvaise haleine, étape du diagnostic, se fait par 2 méthodes [16]. Les méthodes organoleptiques, très pratiques, sont très faciles à utiliser et ne nécessitent aucun matériel; mais elles restent tributaires de l’appréciation subjective de l’examinateur [8]. De plus, l´utilisation de ces scores organoleptiques, est l´étalon-or pour la mesure des mauvaises odeurs orales [16]. Ces échelles organoleptiques sont largement utilisées dans la recherche d´haleine. D’autres méthodes plus objectives de diagnostic de la mauvaise haleine mesurent les composés volatils à la source de l’halitose [8]. Ces derniers sont détectés par une méthode électrochimique (halimètre) détectant une partie des composés sulfurés volatils ou par un procédé de chromatographie en phase gazeuse, plus précis mais bien plus coûteux [8]. Dans notre contexte où la pratique médicale est caractérisée par la rareté des ressources et l’absence des équipements médicaux pour les mesures quantitatives, nous avons opté pour la méthode organoleptique. Toutefois elle était pratiquée par un même praticien, formé à cette méthode diagnostique et indemne de trouble olfactif permettant ainsi de limiter la subjectivité liée à cette technique diagnostique. Au terme de notre démarche diagnostique de l’halitose, l’origine bucco-dentaire était la principale cause de l’halitose avec près de 60% des cas; et l’existence d’une carie dentaire était positivement corrélée à l’intensité de l’halitose. Nos résultats sont en accord avec ceux de la littérature rapportant que la principale cause de l’halitose est d’origine bucco-dentaire avec près de 80-90 % des cas d’halitose [8, 16-19]. Aussi, pour Aka et al. en Côte d’Ivoire, les causes locales (notamment les caries dentaires, les gingivites, les parodontites) représentaient 80-85% des causes d’halitose, concordants avec nos données [3]. De même, plusieurs études transversales ont associée l’halitose soit à la présence d’une gingivite ou d’une parodontite [15,16], similaires aux nôtres. Enfin, des études in vitro et in vivo ont démontré la capacité des agents pathogènes dentaires, parodontaux et muqueux (dont ceux de la face dorsale de la langue) à produire des composés sulfurés volatils (CSV) [14].

Cependant des étiologies non buccales de l’halitose, nettement moins fréquentes, sont également évoquées; ce sont essentiellement des affections oto-rhino-laryngologiques et gastroentérologiques [17]. Plusieurs études antérieures ont suggéré que les maladies du tractus gastro-intestinal (TGI) peuvent causer l´halitose [10,17,20]. Et des études récentes suggèrent que l´infection à *H. pylori* chez les patients atteints de maladies du TGI serait l´une des principales étiologies de l´halitose [10,20]. En effet, lorsque l’estomac se remplit, les glandes gastriques entrent en action pour faciliter la digestion des aliments par la production de chyme [2]. Cependant, certains troubles ou affections (exemple: infection, inflammation, trouble fonctionnel ou anatomique) peuvent perturber ce processus de digestion et engendrer une halitose [2]. *H. pylori* retrouvée dans la muqueuse gastrique, est le facteur causal majeur de la gastrite chronique et de l’ulcère gastrique et/ou duodénal, en accord avec nos données où l’étude histologique rapportait une gastrite chronique chez tous les patients porteurs de la bactérie *H. pylori* [2]. Un lien possible entre H. pylori et l´halitose a été suggéré pour la première fois en 1985 [10]; par la suite le lien entre l’halitose et l’infection par *H. pylori* a été prouvé par plusieurs études cliniques [21-23]. Il a été ainsi établi que si les principaux facteurs de risque locaux tels que la carie dentaire et les maladies parodontales étaient exclus chez des patients présentant une halitose, l´éradication de *H. pylori* aurait pour effet de diminuer significativement voire d’éliminer le taux de CSV (et donc de l’intensité de la mauvaise haleine) [22]. Plus récemment, Hajifattahi et al. rapportaient une association significative entre *H. pylori* et l’halitose dans une étude de cas témoins où les cas étaient des patients présentant une halitose sans cause bucco-dentaire évidente menée en Iran [10]. Bien que la recherche de *H. pylori* n’a pas été faite chez tous nos patients (absence de moyens financiers), nos résultats ont montré une forte fréquence de l’infection au cours de l’halitose (sur 10 patients atteints d’halitose dépistés pour l’infection à *H. pylori*, 90% étaient porteurs de la bactérie), confortant ainsi le lien supposé entre *H. pylori* et l’halitose retrouvé dans la littérature [10, 21-23]. Mais les études existantes montrent des résultats contradictoires; pour d´autres études l´halitose serait liée aux maladies du TGI autres que l´infection à *H. pylori* [20,24]. Moshkowitz et al. ont en effet rapporté une forte association entre la survenue et la sévérité de l’halitose avec le reflux gastro-œsophagien (RGO) [20] et l’absence de lien entre d’autres facteurs de troubles dyspeptiques tels l’ulcère peptique ou l’infection à *H. pylori* et l’halitose [20, 25]. Une étude récente sur des patients présentant une pathologie gastrique a démontré que l´halitose provenait presque toujours de la cavité buccale et non l´estomac [25]. En somme, notre étude en accord avec la littérature suggère un lien entre *H. pylori* et l’halitose même si le mécanisme en cause n’est pas clairement défini [10,17,21-23]. Cette association s’expliquerait par le fait que *H. pylori* contribuerait au développement de l’halitose par une augmentation de la production de CSV [26].

Concernant les pathologies ORL incriminées dans notre étude, la sinusite était associée à la durée des symptômes de l’halitose. Nos résultats s’expliqueraient par la susceptibilité des pathologies de la sphère ORL de générer une halitose avec comme dénominateur commun la présence des CSV véhiculés par la respiration oro-nasale [8]. Ces composés confèrent le caractère malodorant à la respiration, et seraient produits soit par les bactéries impliquées dans un processus infectieux de la sphère ORL, soit par la dégradation des protéines de nature endogène (processus de nécrose tissulaire) ou exogène (processus de putréfaction des détritus alimentaires) [8]. Les sinusites et l’amygdalite retrouvées dans notre étude sont en accord avec les pathologies de la sphère ORL en lien avec l’halitose retrouvées dans d’autres études [3, 8].

Les solutions thérapeutiques, multiples et le plus souvent curatives, dépendent de la cause incriminée. Au plan thérapeutique, le bon taux de satisfaction des patients après deux semaines de traitement s’expliquerait par le nombre élevé de traitement à visée étiologique démontrant ainsi la nécessité d’une recherche étiologique de l’halitose. Nos résultats pourraient être améliorés par l’incitation des services concernés par l’halitose au sein de notre structure hospitalière (odonto-stomatologie, gastro-hépato-entérologie, O.R.L et psychiatrie) à plus de collaboration pour une prise en soins multidisciplinaire de l’halitose. Cela pourrait aboutir à la mise en place d’un parcours de soin bien coordonné pour l’halitose avec le service bucco-dentaire (odonto-stomatologie) comme porte d’entrée comme c’est le cas à la clinique universitaire de médecine dentaire de l’université de Bâle [27].

## Conclusion

Notre étude confirme qu’en plus des affections bucco-dentaires, les atteintes du TGI sont également une source d’halitose dans notre contexte et la fréquence élevée de l’infection à *H. pylori* chez les patients présentant une halitose conforterait le lien entre l’halitose et cette bactérie. Cependant des études utilisant des méthodes diagnostiques plus précises couplées à une recherche systématique de l’infection à *H. pylori* sont nécessaires pour confirmer ce lien et ouvrir une voie pour la recherche de possibles mécanismes de cette association. Aussi, vu le coût de l’halitose entièrement à la charge du patient la rationalisation du parcours de soin de l’halitose (avec le chirurgien-dentiste comme acteur de santé central devant collaborer au sein d’une équipe médicale pluridisciplinaire) s’avère indispensable. Enfin, l’amélioration de la prise en soins de l’halitose passe par l’acquisition de matériels d’explorations objectives de l’halitose (halimètre ou chromatographie en phase gazeuse).

### Etat des connaissances actuelles sur le sujet

- L’halitose est une affection méconnue qui présente à la fois un aspect pathologique et social (maladie honteuse et taboue);
- En pratique clinique, l’halitose pose de nombreux problèmes de prise en charge aux plans diagnostiques et thérapeutiques;
- L’origine buccale représente 85% des causes d’halitose.

### Contribution de notre étude à la connaissance

- Premier travail sur l’halitose et surtout pluridisciplinaire dans notre pays;
- Contribution aux données cliniques étiologiques (rares) sur l’halitose en Afrique.

## Conflits d’intérêts

Les auteurs ne déclarent aucun conflit d’intérêts.
